# Ladinin 1 Shortens Survival via Promoting Proliferation and Enhancing Invasiveness in Lung Adenocarcinoma

**DOI:** 10.3390/ijms24010431

**Published:** 2022-12-27

**Authors:** Chao-Yuan Chang, Yung-Chi Huang, Hung-Hsing Chiang, Yu-Yuan Wu, Kuan-Li Wu, Yung-Yun Chang, Lian-Xiu Liu, Ying-Ming Tsai, Ya-Ling Hsu

**Affiliations:** 1 Graduate Institute of Medicine, College of Medicine, Kaohsiung Medical University, Kaohsiung 807, Taiwan; 2 Department of Anatomy, Kaohsiung Medical University, Kaohsiung 807, Taiwan; 3 Drug Development and Value Creation Research Center, Kaohsiung Medical University, Kaohsiung 807, Taiwan; 4 Division of Thoracic Surgery, Department of Surgery, Kaohsiung Medical University Hospital, Kaohsiung Medical University, Kaohsiung 807, Taiwan; 5 School of Medicine, College of Medicine, Kaohsiung Medical University, Kaohsiung 807, Taiwan; 6 Division of Pulmonary and Critical Care Medicine, Kaohsiung Medical University Hospital, Kaohsiung Medical University, Kaohsiung 807, Taiwan; 7 Division of General Medicine, Kaohsiung Medical University Hospital, Kaohsiung Medical University, Kaohsiung 807, Taiwan

**Keywords:** B3GNT3, EMT, LAD1, lung cancer, migration, proliferation

## Abstract

Lung cancer is one of the deadliest cancers worldwide, including in Taiwan. The poor prognosis of the advanced lung cancer lies in delayed diagnosis and non-druggable targets. It is worth paying more attention to these ongoing issues. Public databases and an in-house cohort were used for validation. The KM plotter was utilized to discover the clinical significance. GSEA and GSVA were adopted for a functional pathway survey. Molecular biological methods, including proliferation, migration, and the EMT methods, were used for verification. Based on public databases, the increased expression of Ladinin 1 (LAD1) was presented in tumor and metastatic sites. Furthermore, an in-house cohort revealed a higher intensity of LAD1 in tumor rather than in normal parts. The greater the expression of LAD1 was, the shorter the duration of lung adenocarcinoma (LUAD) patient survival. Moreover, the association of B3GNT3 with LAD1 affected the survival of LUAD patients. Functional analyses using GSEA and GSVA revealed the associations with survival, migration, invasion, and EMT. Biologic functions supported the roles of LAD1 in proliferation via the cell cycle and migration in EMT. This study reveals that LAD1 plays a major role in regulating proliferation and migration in lung cancer and impacts survival in LUAD. It is worth investing in further studies and in the development of drugs targeting LAD1.

## 1. Introduction

According to the World Health Organization, lung cancer ranks first worldwide in terms of cancer deaths [1]. Non-small cell lung cancer, especially adenocarcinoma, is the most prevalent histologic subtype of lung cancer [2], followed by squamous cell carcinoma and large cell carcinoma. Small cell lung cancer is distinct from non-small cell lung cancer accounting for 10–15% of total lung cancer cases. The prognosis of lung cancer is poor, which may be partly explained by its delayed diagnosis. About 70% of lung cancer patients are diagnosed in the late stages with extensive metastases [3,4]. For decades, much work has been undertaken to improve the outcome of lung cancer patients. Successful strategies include the early detection of lung cancer by implementing a low-dose computed tomography screening program [5], the identification of actionable genetic mutations accompanied by the use of corresponding targeted agents, and the discovery and application of immune therapies [6,7]. Though survival is prolonged somewhat by the above efforts, optimal and effective treatment of lung cancer still has a long way to go. Further investigations are urgently needed to search for more therapeutic options for lung cancer.

Ladinin 1 (LAD1) is a protein-coding gene of approximately 19,000 base pairs that is located on chromosome 1. The translated ladinin 1 protein is composed of 517 amino acids, with an approximate molecular weight of 57 kDa. Ladinin 1 is often expressed in squamous and glandular epithelia with a predominant localization on the cell membrane as well as in the cytoplasm [8]. The best-known function of LAD1 is that it is the anchoring filament protein, which is a component of the basement membrane zone. The formation of autoantibodies against LAD1 may lead to autoimmune diseases such as bullous dermatosis, manifesting as blisters resulting from disrupted basement membranes [9]. However, the dysregulation of LAD1 is also involved in malignancy. LAD1 is associated with progressive behaviors and is linked to poor outcomes in various cancer types. For example, transcriptomic studies have demonstrated that LAD1 mRNA is expressed at higher levels in tumor tissue and that its up-regulation is correlated with poor survival in breast cancer [10], colorectal cancer [11], and prostate cancer [12]. In pancreatic cancer, the higher expression of LAD1 is also linked to resistance to chemotherapeutic agents [12]. Regarding the promoting effect of cancer, Lad1 is reported to enhance the migration and invasion of colorectal cancer cells and facilitate further metastatic progression in a mouse model [11].

Transcriptomic and proteomic studies have demonstrated that LAD1 is also abundant in lung adenocarcinoma [13,14]. Moreover, higher expression levels of LAD1 in lung adenocarcinoma also confers a poor prognosis [14]. In vitro, A549 and PC9 cells have been shown to be more viable and invasive due to the overexpression of LAD1, which is secondary to the inhibition of miR-331 [14].

Collectively, the reported literature suggests that the role of LAD1 in the aggressive progression of various cancers is accompanied by the upregulation of LAD1. Nonetheless, the molecular function of LAD1 in the progression of lung cancer remains elusive. In addition, studies assessing the involvement of LAD1 in lung cancer progression are lacking. Therefore, the present study aims to survey the expression pattern, the associated functional pathways, and possible regulatory mechanisms of LAD1 in lung adenocarcinoma. Cell behaviors promoted by LAD1 are also being investigated in vitro.

## 2. Results

### 2.1. LAD1 mRNA Expression and Its Association with Mutated Genes in LUAD

**Figure 1:**
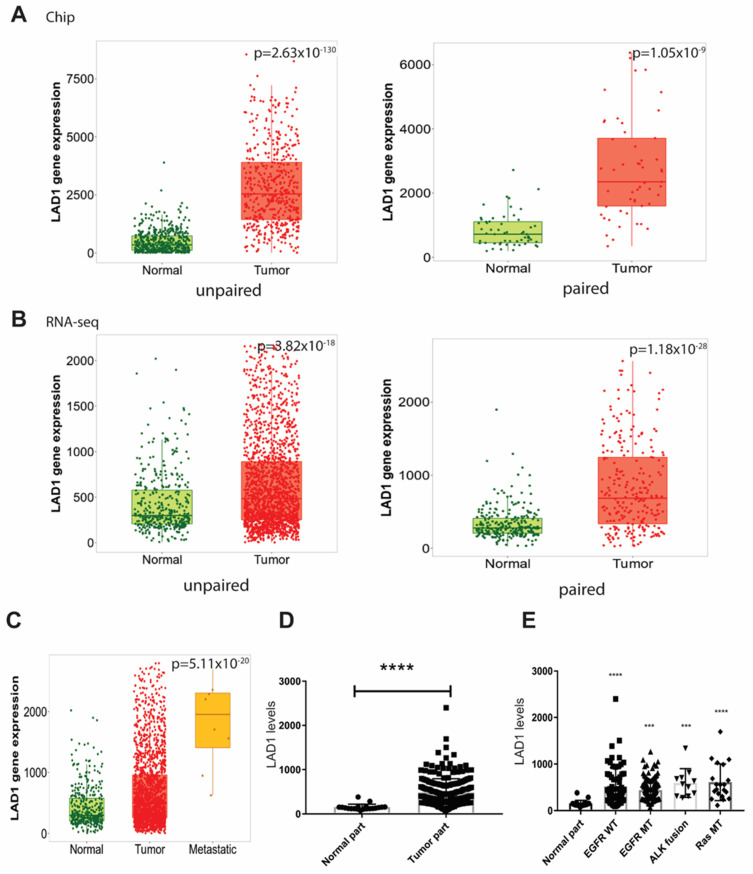
The mRNA expression of LAD1 in lung adenocarcinoma (LUAD) and its association with targetable mutated gene. (**A**) LAD1 expression in un-paired normal tissue and tumor (left) or paired tissue (right) of LUAD using gene chip data or (**B**) RNA−seq data from TNMplot website. All express at significantly higher levels in tumor parts than in normal tissues. (**C**) Comparison of the expression of LAD1 in normal, tumor, and metastatic tissues from gene chip data. (**D**) LAD1 expression in tumor and normal parts of pathological stages I to II lung adenocarcinoma from GSE31210. (**E**) Expression levels of LAD1 were significantly higher in tumors without epithelial growth factor receptor mutation (EGFR WT) and with EGFR mutation (EGFR MT), ALK fusion (ALK fusion), and KRAS mutation (RAS MT) than in normal parts from GSE31210. Green, normal parts; red, tumor parts; yellow, metastatic parts. (***, *p* < 0.005; ****, *p* < 0.001).

The analysis of LAD1 mRNA expression in the chip database (Figure 1A) and gene RNA-seq database (Figure 1B) of lung adenocarcinoma (LUAD) patients through the TNMplot showed that LAD1 was expressed significantly at higher levels in the tumor tissue than in the normal one when compared with either paired or unpaired methods. Upon subdividing the tumor tissues into normal, tumor, and metastatic tissue from the gene chip database, it was found that LAD1 expression was significantly upregulated in the metastatic tissues (Figure 1C). Similarly, the expression of LAD1 was also profoundly upregulated in the tumor parts of pathological stages I to II LUAD from the GSE31210 database (Figure 1D). We further dissected the association of LAD1 expression with targetable mutated genes using the GSE31210 database. An increase in LAD1 expression was not correlated with an epithelial growth factor receptor (EGFR) mutation (Figure 1E). Similarly, LAD1 was also upregulated when ALK fusion or KRAS mutation (RAS MT) was detected (Figure 1E) (*p*-value < 0.005). These results suggest that the overexpression of LAD1 in the tumor parts of LUAD patients is concomitant with ALK and RAS mutations, at the exclusion of the EGRF mutation.

### 2.2. LAD1 Protein Expression Level in LUAD

**Figure 2:**
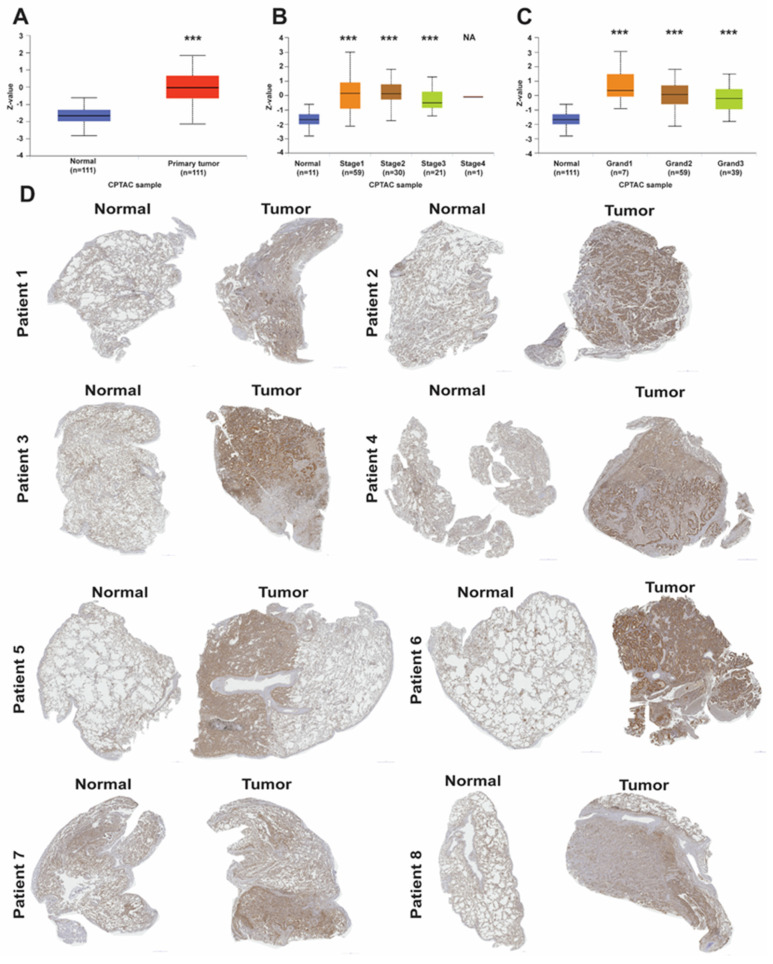
The protein expression of LAD1 in LUAD. (**A**) LAD1 protein had significantly lower expression levels in normal tissues than in the primary tumors in LUAD from the CPTAC cohort. The protein expression of LAD1 was significantly increased among the tumor stages (**B**) and the tumor grades (**C**) in the CPTAC cohort. The Z-values represent standard deviations from the median across samples for lung cancer. (**D**) Comparison of LAD1 protein expression between paired normal tissues (left) and tumor tissues (right) from the eight in-house LUAD patients by immunohistochemistry (IHC) staining. (***, *p* < 0.005).

Along with LAD1 mRNA expression, LAD1 protein expression levels in the primary LUAD tumor were significantly increased in the CPTAC cohort (*p*-value < 0.001) (Figure 2A). Compared with expression levels in normal tissues, LAD1 protein was significantly overexpressed in LUAD patients in stages I, II, and III (*p*-value < 0.001) but was not stage-dependent (Figure 2B). As for the appearance of cancer cells, known as cancer grades, LAD1 protein levels were also significantly higher in all the cancer grades than in the normal tissue (*p*-value < 0.001) (Figure 2C). Furthermore, the protein expression of LAD1 in paired normal and tumor tissues from the eight in-house patients was inspected with immunohistochemistry staining, and all eight of the samples overexpressed LAD1 proteins in their tumor parts (Figure 2D). These data suggest that LAD1 is upregulated in LUAD.

### 2.3. The Survival Significance of LAD1

**Figure 3:**
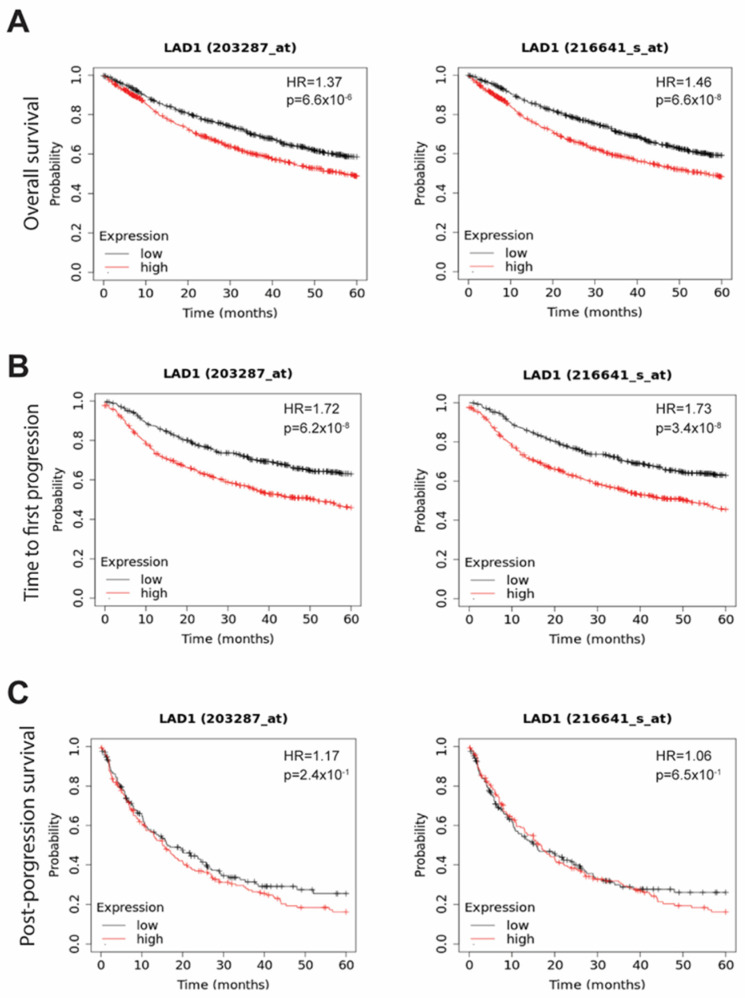
Five-year survival analysis of LAD1 expression in LUAD. (**A**) Poor outcomes of five-year overall survival, time to first progression (**B**), and post-progression survival (**C**) are associated with high LAD1 expression levels identified by the Kaplan−Meier plotter (KM plotter).

After recording the overexpression of LAD1 mRNA and protein in tumor parts, we explored its prognostic role in lung cancer using the KM plotter. The five-year overall survival (OS) (Figure 3A) outcomes of LUAD patients with upregulated LAD1 mRNA expression were worse in both probe sets (Affymetrix ID 203287_at and 216641_s_at) (n = 1925, *p*-value < 0.001). Similarly, the time to first progression (FP) (Figure 3B) was shorter when LAD1 expression levels were high (n = 982, *p*-value < 0.001) (Figure 3B). On the contrary, LAD1 expression was not correlated with post-progression survival (PPS) (n = 344, *p*-value > 0.05) (Figure 3C). It is suggested that LAD1 expression is negatively correlated with OS and FP in lung cancer patients.

### 2.4. The Functional Role and Pathways Associated with LAD1

**Figure 4:**
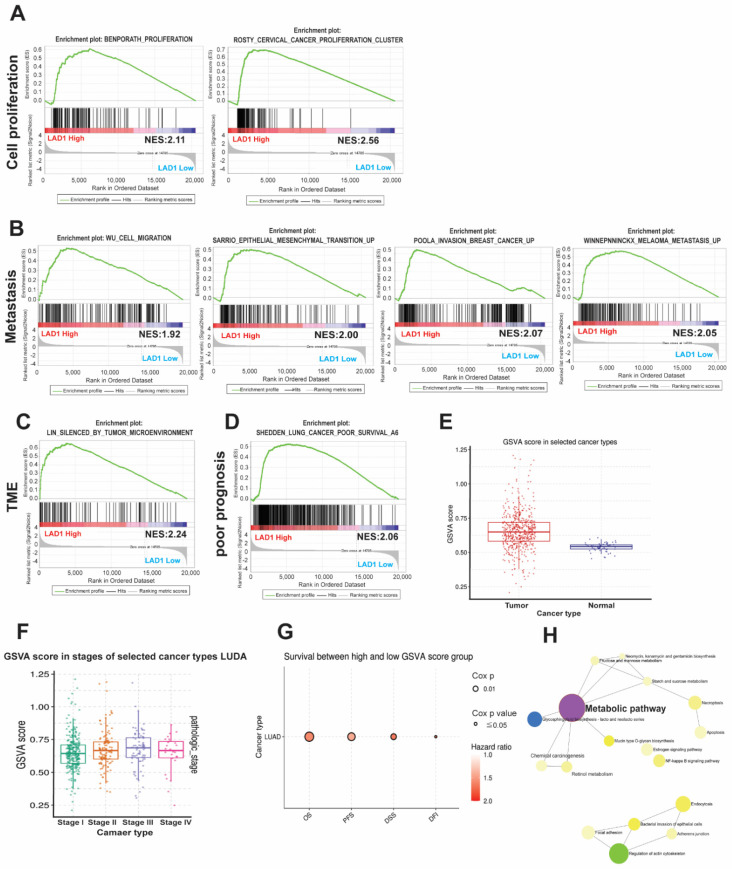
Functional status and pathway analysis of LAD1 in LUAD. (**A**) The association of cell proliferation, tumor metastasis (**B**), the tumor microenvironment (**C**), and poor prognosis (**D**) with LAD1 expression is analyzed by gene set enrichment analysis (GSEA). All these functional statuses are all enriched with high LAD1 expression. Meanwhile, gene set variation analysis (GSVA) scores were also used to analyze LAD1 expression difference between the tumor tissues and normal tissues (**E**), among the caner stages (**F**), and the survival analysis (**G**) from the TCGA datasets. (**H**) The pathways positively correlated with LAD1 according to correlated gene sets are illustrated. The metabolism pathway is significantly associated with LAD1 expression in LUAD.

To dissect the functional role of LAD1 in cancer progression, we identified the association of LAD1 with cancers using two enrichment analyses, GSEA and GSVA. The GSEA results showed that increased LAD1 expression was positively associated with cell proliferation (Figure 4A), cancer cell metastasis (Figure 4B), and the tumor microenvironment (Figure 4C) in cancers. Compatible with the survival analysis in lung cancer, the GSEA revealed that high expression levels of LAD1 were correlated with a poor prognosis in LUAD (Figure 4D). On the other hand, we calculated the GSVA score of LAD1 positively correlated gene sets to explore functional pathways using the GSCA website. The GSVA scores were higher in tumor parts (Figure 4E) and in advanced cancer stages (Figure 4F). Meanwhile, the GSVA score of LAD1 positively correlated gene sets were significantly associated with poor outcomes, including overall survival (OS, HR = 1.59, Cox *p*-value = 0.001), progression-free survival (PFS, HR = 1.43, Cox *p*-value = 0.003), disease-specific survival (DSS, HR = 1.65, Cox *p*-value = 0.008), and disease-free survival (DFS, HR = 1.68, Cox *p*-value = 0.014) (Figure 4G). The annotation of correlated pathways with these LAD1-associated gene sets showed that the metabolic pathway was the most significant (Figure 4H). These bioinformatic analysis reports suggest that upregulation of LAD1 is strongly associated with cancer progression and poor clinical outcomes, mainly through a metabolic pathway.

### 2.5. The Impact of LAD1 Correlated Metabolic Pathway Genes

**Figure 5:**
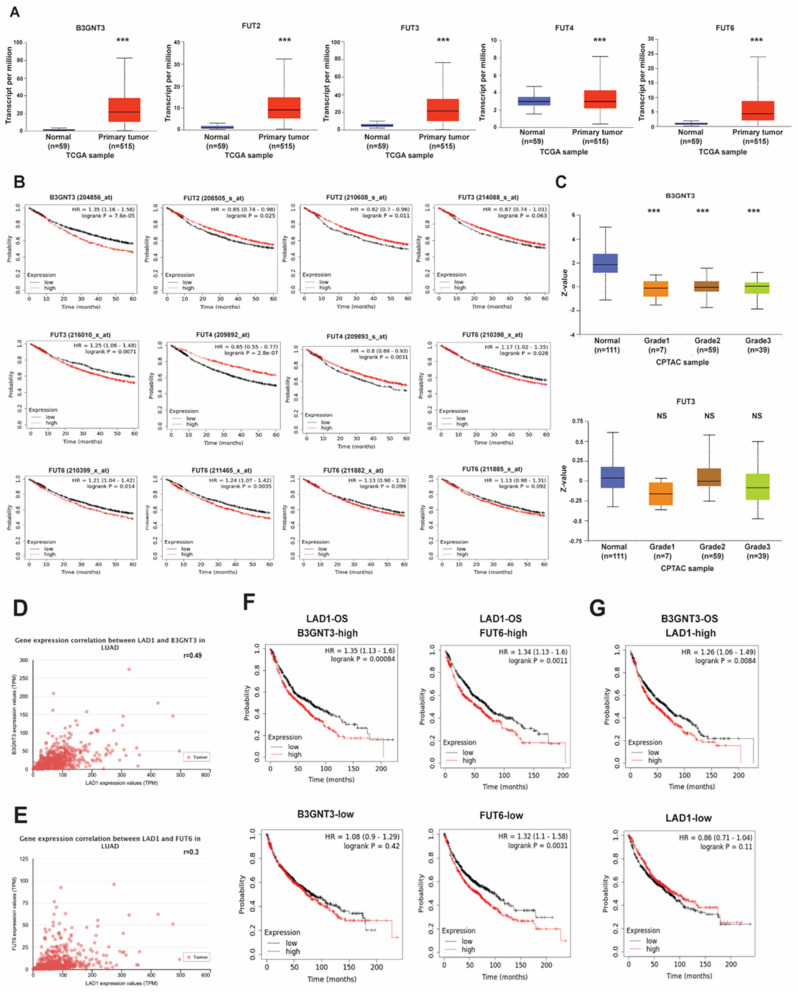
Correlation of metabolism associated genes with LAD1 expression in LUAD. (**A**) Metabolism−associated genes, including B3GNT3, FUT2, FUT3, FUT4, and FUT6, were expressed at higher levels in the primary tumor than in the normal parts in LUAD in the TCGA datasets. (**B**) Overall survival (OS) of these genes analyzed by the KM plotter. (**C**) The protein expressions of B3GNT3 and FUT3 among cancer grades in LUAD were shown in the CPTAC cohorts. (**D**) LAD1 expression is positively correlated with B3GNT3 and FUT6 (**E**) in the TCGA datasets. (**F**) Cross-analysis between either B3GNT3 or FUT6 and LAD1 in survival. (**G**) Poor overall survival links to high B3GNT3 expression levels only when LAD1 expressed high levels in LUAD. (ns, not significant; ***, *p* < 0.005).

Based on the previous GSVA, five LAD1 and metabolic pathway-associated genes were selected, including B3GNT3, FUT2, FUT3, FUT4, and FUT6. All five of the genes were expressed at higher levels in the primary tumor than in the normal tissue of the LUAD patients from the TCGA cohort (Figure 5A). However, the increased expression of B3GNT3 in only one probe set, FUT2 and FUT4 in none, FUT3 in one of two probe sets, and FUT6 in three out of five probe sets were significantly associated with poor survival (Figure 5B). B3GNT3 protein expression was significantly correlated with tumor grading in lung cancer cells but not FUT3 (the only two available data from CPTAC) (Figure 5C). The Pearson correlation coefficients of B3GNT3 and FUT6 expression with LAD1 were 0.49 and 0.3, respectively, on the UALCAN website, which means these three genes were statistically correlated in LUAD patients (Figure 5D,E). Cross-analysis revealed that LAD1 overexpression was significantly associated with poor outcomes only when B3GNT3 expression increased. However, OS was negatively correlated with LAD1 expression regardless of the FUT6 expression levels (Figure 5F). On the other hand, the low expression of B3GNT3 was a statistically significant better prognostic factor when LAD1 expression increased (Figure 5G). It suggests that concurrent overexpression of B3GNT3 and LAD1 strongly predicts a poor prognosis.

### 2.6. The Functional Role of B3GNT3 and Its Association with Targetable Mutated Genes

**Figure 6:**
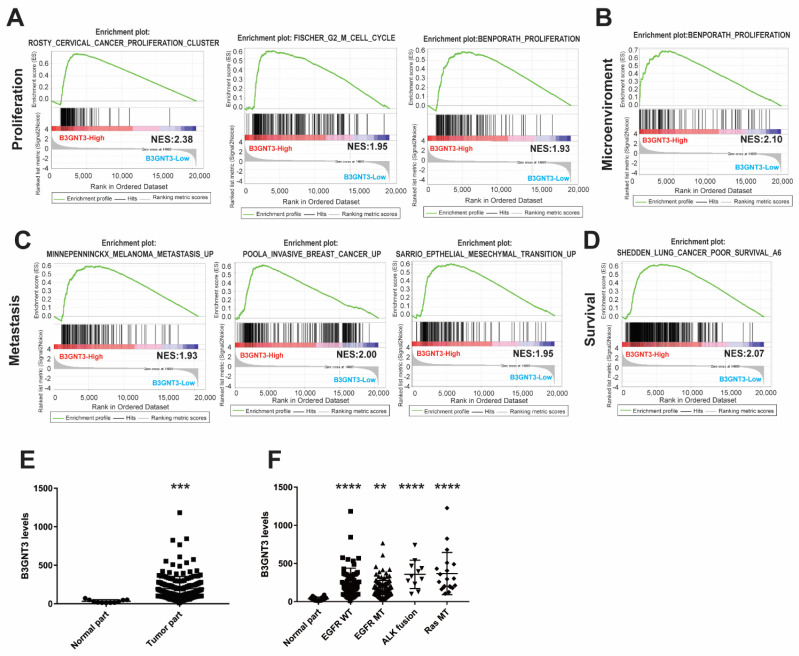
Functional status of B3GNT3 in LUAD and its correlation with targetable mutated genes. (**A**) B3GNT3 expression is positive correlated with cell proliferation, cell cycle and the tumor microenvironment (**B**), tumor metastasis (**C**), and poor prognosis (**D**) based on GSEA. (**E**) B3GNT3 expression in tumor and normal parts of LUAD from GSE31210 dataset. (**F**) The expression of B3GNT3 in normal tissues and in tumors without epithelial growth factor receptor mutation (EGFR WT), with EGFR mutation (EGFR MT), ALK fusion (ALK fusion), and KRAS mutation (RAS MT) are listed, taken from the GSE31210 dataset. (**, *p* < 0.01; ***, *p* < 0.005; ****, *p* < 0.001).

The GSEA findings showed that upregulated B3GNT3 was statistically correlated with cell proliferation, cell cycle, cancer metastasis, and EMT in cancers (Figure 6A–C). As with LAD1, poor clinical outcomes were significantly associated with increased B3GNT3 expression in lung cancer patients (Figure 6D). Meanwhile, B3GNT3 expression was significantly upregulated in the tumor parts compared with its expression in the normal ones in the GSE31210 database (Figure 6E). However, there were no statistical differences in B3GNT3 expression between normal tissues and tumors with mutated genes, such as EGFR, ALK, and RAS. Interestingly, the expression of B3GNT3 in wild-type EGFR tumors differed significantly from that in EGFR-mutated tumors in LUAD patients (Figure 6F). These data showed that B3GNT3 is overexpressed in tumor parts, especially in tumors without an EGFR mutation. B3GNT3 is also associated with tumor progression.

### 2.7. Functional Analysis of Knockdown LAD1 In Vitro

**Figure 7:**
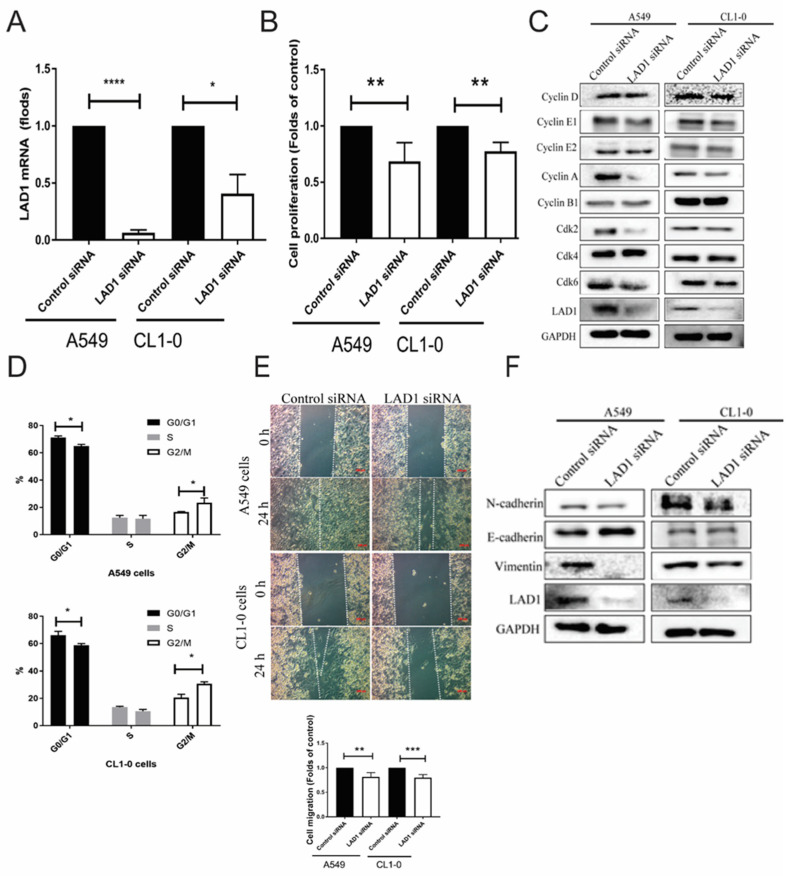
Functional analysis of LAD1 in LUAD. (**A**) Knockdown efficiency of LAD1 via siRNA method. (**B**) Cell proliferation is counted when LAD1 expression was downregulated with in A549 and CL1-0 LUAD cancer cell lines. (**C**) Cell cycle associated proteins are analyzed by Western blot. (**D**) Cell cycle analysis using a flow cytometry. (**E**) Cell migration in LAD1 knockdown A549 and CL1-0 cell line is assessed by wound-healing assay. (**F**) Epithelial mesenchymal transition (EMT) markers are also evaluated with Western blot. Both A549 and CL1-0 LUAD cancer cell lines are transfected with control siRNA or LAD1 siRNA. All experiments were performed independently at least three times. (*, *p* < 0.05; **, *p* < 0.01; ***, *p* < 0.005; ****, *p* < 0.001).

The role of LAD1 in cancer progression was validated by the A549 and CL1-0 LUAD cancer cell lines transfected with control siRNA or LAD1 siRNA with effective knockdown (Figure 7A). When LAD1 was knockdown by LAD1 siRNA, cell proliferation decreased in both cell lines (Figure 7B). Therefore, this study analyzed cell cycle-associated proteins, including cyclins and cyclin-dependent kinases (Cdk), to explore the functional roles of LAD1. When LAD1 was knocked down, cyclin E1, E2, A, and Cdk2 expression was also decreased in both the A549 cells and the CL1-0 cells, suggesting the inhibition of the synthesis phase, whereas there was no change to the cyclin D, B, Cdk4, or Cdk6 expression (Figure 7C). The cell cycle analysis revealed cells arrested at the G2/M phase (Figure 7D), which is compatible with the protein pattern in Figure 7C. The proliferation was not related to apoptosis by apoptotic analysis (Figure S1). The wound-healing assay also showed a delayed closure process in LAD1 knockdown A549 and CL1-0 cells (Figure 7E). Apart from cell proliferation, the A549 cells and CL1-0 cells knocked down by LAD1 siRNA shifted from the mesenchymal (N-cadherin and vimentin) to the epithelial (E-cadherin) phenotype (Figure 7F). These results hint that lower levels of LAD1 are associated with a less invasive phenotype in LUAD.

## 3. Discussion

Lung cancer has been the leading cause of cancer death worldwide for decades, accounting for approximately 25% of all cancer deaths. The current treatment strategies for lung cancer are palliative, and the responses to current standard therapies are poor. A better understanding of the pathology of lung cancer might lead to the development of more efficacious and specific drugs. Our study indicates that LAD1 acts as an oncogene in lung cancer development. Clinically, LUAD patients with high levels of LAD1 carry poor outcomes. Furthermore, LAD1 potentiates oncogenesis in LUAD by regulating proliferation, migration, and epithelial-mesenchymal transition (EMT). This study provides evidence that LAD1 is a potential target for developing therapeutic agents for lung cancer.

LAD1 has been reported as being abundant in various cancers, including LUAD [13,14]. The expression of LAD1 is regulated by miR-331, which is downregulated in A549 and PC9 lung cancer cells [14]. In this study, we found that the expression of LAD1 was increased in the tumors of LUAD compared with that in normal tissues, and the expression of LAD1 was further increased in metastatic tumors. The survival analyses from the KM plotter revealed lower OS and FP, but this was not the case for PPS in lung cancer patients with high LAD1 expression. The GSEA of the LAD1-correlated gene set strongly associated with cell proliferation, EMT, and metastasis in LUAD indicated that LAD1 lung cancer growth by regulating the cell cycle. Subsequent functional experiments showed that LAD1 knockdown decreased the cell migration of lung adenocarcinoma cells by regulating EMT.

The gene set variation analysis (GSVA) showed that the gene set positively correlated with LAD1 level and is associated with poor prognosis, including OS, PFS, DSS, and DFS. The pathway analysis showed that the gene set is involved in the metabolic pathway, which includes Beta-1,3-N-acetylglucosaminyltransferase 3 (B3GNT3) and FUT 2, 3, 4, and 6. To narrow down the candidate genes, we utilized survival analysis, tumor grades, a correlation study, and cross-analysis, B3GNT3 was highly correlated with LAD1. Consistent with the GSEA of LAD1, the transcriptomics of LUAD patients with a high level of B3GNT3 were associated with cancer metastasis, EMT, and poor prognosis. B3GNT3, a member of the β3GlcNAcT gene family, is an enzyme essential for the biosynthesis of poly-N-acetyllactosamine chains and the backbone structure of dimeric sialyl Lewis A, and it plays a significant role in L-selectin ligand biosynthesis. The β3GlcNAcT family members, including B3GNT3, are closely correlated with the development and progression of various cancers. Previous studies have reported the involvement of B3GNT3 in tumorigenesis and that B3GNT3 is strongly associated with PD-L1 expression and EGFR mutation status in lung adenocarcinoma [15]. The expression of B3GNT3 is also highly associated with immune cell infiltration in gynecologic cancers, pancreatic carcinoma, and lung adenocarcinoma [16,17,18]. In our study, the GSEA showed that the transcriptomes of LUAD patients with high levels of LAD1 and B3GNT3 were strongly associated with the tumor microenvironment, suggesting that they cooperated to build the tumor microenvironment. However, the interaction of LAD1 and B3GNT3 is worth further exploration.

## 4. Materials and Methods

### 4.1. Bioinformatics

The unpaired and paired expression of genes between normal lung tissues and lung tumors were extracted from the RNA-seq data and gene chip data of TNMplot website (https://tnmplot.com/analysis/, accessed on 21 June 2022) [19] or The Cancer Genome Atlas (TCGA) using UALCAN website (http://ualcan.path.uab.edu/, accessed on 21 June 2022) [20]. Using these websites, the differential expression pattern of the input gene in tumor or normal parts could be demonstrated. The criteria for the significant differentially expressed genes (DEGs) in the analysis were defined as a fold change (tumor/normal) of expression level > 2 or <0.5 and a *p*-value < 0.05, which was calculated using the UALCAN website.

The Asian cohort GSE31210 dataset from the Gene Expression Omnibus (GEO, https://www.ncbi.nlm.nih.gov/geo/query/acc.cgi?acc=GSE31210 accessed on 21 June 2022) was used to validate the results from TNMplot website. In GSE31210 dataset, the enrolled patients were further subgrouped into those were EGFR mutant, ALK mutant, RAS mutant, triple-negative mutant, and normal [21]. The gene expression pattern was also analyzed in the various mutation groups.

The protein expression level was extracted from the CPTAC lung adenocarcinoma cohort via the UALCAN website. Using this website, the protein-expressing levels in tumor or normal parts, as well as in different extent of lymph node involvement and cancer stages could be demonstrated.

### 4.2. Survival Analysis

The KM plotter database (http://kmplot.com/analysis/, accessed on 19 July 2022) was used to analyze the association of the mRNA expression with overall survival (OS), time to first progression (FP) and post-progression survival (PPS) [22]. Patients were divided into 2 groups by the best cut-off value of gene expression. The survival probability in a 60-month period of both high- and low-expression groups were plotted by Kaplan–Meier method and compared by log-rank test. The hazard ratios (HR) with 95% confidence intervals (CI) and *p*-values were extracted from the KM plotter webpage and considered significant with *p*-values < 0.05.

### 4.3. Immunohistochemistry

The pairs of normal lung tissues and tumors were harvested from the Division of Thoracic Surgery and Division of Pulmonary and Critical Care Medicine, Kaohsiung Medical University Hospital (Kaohsiung, Taiwan, KMUH-IRB-20180023, KMU-IRB-20200038; KMU-IRB-E(II)-20220175). LAD1 antibody (Abcam, UK, cat no.: ab24688) was used for the immunohistochemistry stain.

### 4.4. Gene Set Enrichment Analysis (GSEA)

To explore the functional states of selected gene set, the Gene Set Enrichment Analysis (GSEA) is developed and available online that computes the correlations between the pre-defined gene set and its biological functions or pathological states [23]. In this study, the gene expression profile of TCGA LUAD cohort was divided into LAD1 high- and low- groups defined as the 1st and 4th quartiles. Thereafter, GSEA was conducted to analyze the enrichment score of the gene set expression between high- and low-LAD1 groups. False discovery rate (FDR) < 0.05 and a *p*-value < 0.05 were defined as the cutoff value. The gene set “c2.cp.kegg.v6.2.symbols.gmt” was chosen as the reference.

### 4.5. Gene Set Variation Analysis (GSVA) and Metascape

First, the positively or negatively correlated gene sets with LAD1 were extracted from the UALCAN website with the following criteria: Pearson-CC (correlation coefficient) > 0.3 and *p*-value < 0.05. GSVA (Gene Set Variation Analysis) score of any sample was calculated according to the expression of the given gene set by the GSCA website (http://bioinfo.life.hust.edu.cn/GSCA, accessed on 21 June 2022) [24]. Further comparison of the GSVA score distribution in tumor or normal could be achieved. In addition, the survival associated with the GSVA scores was estimated as well. The positively correlated gene set of LAD1 was also analyzed on the Metascape website [25] for a more comprehensive annotation (Available online: https://metascape.org/gp/index.html#/main/step1, accessed on 23 July 2022). The correlated pathway enriched with LAD1 positively correlated gene list is illustrated as a clustergram.

### 4.6. Functional Assays

The knockdown strategy was used to test the biological role of LAD1 in LUAD. First, A549 and CL1-0 cells were transfected with control siRNA or LAD1 siRNA using ON-TARGET plus SMARTpool siRNA with Dharmafect reagents No1 (Dharmacon, Lafayette, CO, USA). The sequences of control siRNA were UGGUUUACAUGUCGACUAA; UGGUUUACAUGUUGUGUGA; UGGUUUACAUGUUUUCUGA; UGGUUUACAUGUUUUCCUA. The sequences of LAD1 siRNA were GAACCAACCAAGCUAGGAA; CAGUGAAGUUGGGAGAGAA; CCACACGGCCAUACGGAGA; UCAUUUACUCUCAGGUGUA. The knockdown efficiency of LAD1 siRNA was re-confirmed by RT-PCR at post-transfection 24 h and 48 h, respectively. The primers used for LAD1 are the following: forward sequence (5′-3′): CTGGAGAGATACCACACGG; reverse sequence (5′-3′): CAACCCCTGAGAGCCTCAAG

Cell proliferation assay. The cell proliferation study was operated by WST-1 method. The control siRNA or LAD1 siRNA transfected A549 cells and CL1-0 cells were subjected to WST-1 (EMD Millipore, Burlington, MA, USA) for an incubation time of 72-h, according to the manufacturer’s protocol.

The analysis of cell cycle and apoptosis. For cell cycle analysis, control siRNA and Lad1 siRNA transfected cells were fixed in chilled 70% ethanol, followed by staining with 50 μg/mL propidium iodide (PI) at room temperature for 20 min before analysis. The distribution of cell cycle was assessed by BD Accuri™ C6 Plus Flow Cytometer (BD Biosciences, USA). For apoptosis assay, control-siRNA or LAD1-siRNA transfected cells were harvested after transfection and then processed to stain with Annexin V-FITC Early Apoptosis Detection Kit (Cell signaling technology, #6592) according to the manufacturer’s instructions. The flow cytometry analysis was performed by BD Accuri™ C6 Plus Flow Cytometer (BD Biosciences, USA). Results were representative of 3 independent experiments. *Wound-Healing Assay. LAD1* siRNA or control were transfected into A549 and CL1-0 cells, respectively, and then were seeded in the 24-well plates, which were allowed to grow into 90% of confluence. Equal scratch lines were made through the center of the well using a micropipette tip, followed by washing once with phosphate-buffered saline (PBS) on the following day. Photographs of the wound-healing processes at 0 h and 24 h were captured by Olympus inverted microscope.

Western blotting assay. The total protein of LAD1-knockdown (by siRNA) and control A549 and CL1-0 cells were extracted using the radio-immunoprecipitation assay (RIPA) (EMD Millipore, Billerica, MA, USA) supplemented by a protease inhibitor cocktail (Sigma-Aldrich, St. Louis, MO, USA). An equal volume of total protein was denatured by heat and then separated by a sodium dodecyl-sulfate polyacrylamide gel electrophoresis. Proteins in the gel were transferred onto polyvinylidene difluoride membranes (EMD Millipore, Billerica, MA, USA) by electroblotting, which was probed with primary antibodies overnight after blocking in 5% nonfat dry milk/TBST, followed by incubation with horseradish peroxidase (HRP)-conjugated secondary antibodies (Cell-Signaling Technology, Danvers, MA, USA). The signal of the specific protein was detected using a chemiluminescence kit (EMD Millipore, Billerica, MA, USA). For the investigations of cell cycle progression and epithelial-mesenchymal transition in LUAD, the applied primary antibodies included those against Cyclin A (cat no: #4656), Cyclin B1 (cat no: #4183), Cyclin D (cat no: #2978), Cyclin E1 (cat no: #4129), Cyclin E2 (cat no: #4132), Cdk 2 (cat no: #2546), Cdk 4 (cat no: #2906), and Cdk 6 (cat no: #3136), N-cadherin (catalog#610921), E-cadherin (catalog#610182), and Vimentin (catalog#550513) were purchased from Becton Dickinson biosciences, USA. Anti-LAD1 antibody (cat no. ab246885) was bought from Abcam (UK), and anti-GAPDH (catalog#MAB374) antibodies were from EMD Millipore, Becton Dickinson biosciences. Results of the western blot were quantified by ImageJ software and each experiment was repeated for at least three times independently.

### 4.7. Statistical Analysis

Results were presented as mean ± standard deviation (SD). Between group comparisons were calculated by unpaired Student’s t-test with the assistance of the GraphPad Prism (9.02 version, Graphpad Software, San Diego, CA, USA). Results were considered statistically significant when the *p*-value is less than 0.05.

## 5. Conclusions

Taken together, the results of our study provided evidence that the upregulation of LAD1 and B3GNT3 in LUAD and is a possible mechanism related to the increased mesenchymal phenotype in lung cancer and to the remodeling of the tumor microenvironment. They act as novel and promising prognostic factors in lung cancer. More extensive studies are requested and would be valuable for further confirmation regarding the interaction between LAD1 and B3GNT3 on the progression of lung cancer.

## Data Availability

Gene Expression Omnibus (GEO): https://www.ncbi.nlm.nih.gov/geo/query/acc.cgi?acc=GSE31210 accessed on 28 June 2022; GSCA website: http://bioinfo.life.hust.edu.cn/GSCA accessed on 21 June 2022; KM plotter database: http://kmplot.com/analysis/ accessed on 21 June 2022; Metascape website: https://metascape.org/gp/index.html#/main/step1 accessed on 23 July 2022; TNMplot website: https://tnmplot.com/analysis/ accessed on 21 June 2022; UALCAN website: http://ualcan.path.uab.edu/ accessed on 21 June 2022.

## Supplementary Materials

The following supporting information can be downloaded at: https://www.mdpi.com/article/10.3390/ijms24010431/s1, Figure S1: The effects LAD1 on apoptosis. (A) The status of cell death determined by number of cells stained with annexin V and propidium iodide (PI) using a flow cytometry. (B) Mean rate of apoptosis. All experiments were performed independently at least three times. ns, not significant.

## References

[1] Sung H., Ferlay J., Siegel R.L., Laversanne M., Soerjomataram I., Jemal A., Bray F. (2021). Global Cancer Statistics 2020: GLOBOCAN Estimates of Incidence and Mortality Worldwide for 36 Cancers in 185 Countries. CA Cancer J. Clin.

[2] Li C., Lu H. (2018). Adenosquamous carcinoma of the lung. OncoTargets Ther..

[3] Nicholson A.G., Tsao M.S., Beasley M.B., Borczuk A.C., Brambilla E., Cooper W.A., Dacic S., Jain D., Kerr K.M., Lantuejoul S. (2022). The 2021 WHO Classification of Lung Tumors: Impact of Advances Since 2015. J. Thorac. Oncol..

[4] Luo Y.-H., Liang K.-H., Huang H.-C., Shen C.-I., Chiang C.-L., Wang M.-L., Chiou S.-H., Chen Y.-M. (2022). State-of-the-Art Molecular Oncology of Lung Cancer in Taiwan. Int. J. Mol. Sci..

[5] Bonney A., Malouf R., Marchal C., Manners D., Fong K.M., Marshall H.M., Irving L.B., Manser R. (2022). Impact of low-dose computed tomography (LDCT) screening on lung cancer-related mortality. Cochrane Database Syst. Rev..

[6] Deb D., Moore A.C., Roy U.B. (2022). The 2021 Global Lung Cancer Therapy Landscape. J. Thorac. Oncol..

[7] Singh N., Temin S., Baker S., Blanchard E., Brahmer J.R., Celano P., Duma N., Ellis P.M., Elkins I.B., Haddad R.Y. (2022). Therapy for Stage IV Non–Small-Cell Lung Cancer Without Driver Alterations: ASCO Living Guideline. J. Clin. Oncol..

[8] Teixeira J.C., de Filippo C., Weihmann A., Meneu J.R., Racimo F., Dannemann M., Nickel B., Fischer A., Halbwax M., Andre C. (2015). Long-Term Balancing Selection in LAD1 Maintains a Missense Trans-Species Polymorphism in Humans, Chimpanzees, and Bonobos. Mol. Biol. Evol..

[9] Cozzani E., Di Zenzo G., Gasparini G., Salemme A., Agnoletti A., Vassallo C., Caproni M., Antiga E., Marzano A., Cavalli R. (2020). Autoantibody Profile of a Cohort of 54 Italian Patients with Linear IgA Bullous Dermatosis: LAD-1 Denoted as a Major Auto-antigen of the Lamina Lucida Subtype. Acta Derm. Venereol..

[10] Roth L., Srivastava S., Lindzen M., Sas-Chen A., Sheffer M., Lauriola M., Enuka Y., Noronha A., Mancini M., Lavi S. (2018). SILAC identifies LAD1 as a filamin-binding regulator of actin dynamics in response to EGF and a marker of aggressive breast tumors. Sci. Signal..

[11] Moon B., Yang S.J., Park S.M., Lee S.H., Song K.S., Jeong E.J., Park M., Kim J.S., Yeom Y.I., Kim J.A. (2020). LAD1 expression is associated with the metastatic potential of colorectal cancer cells. BMC Cancer.

[12] Li J., Wang Z., Tie C. (2021). High expression of ladinin-1 (LAD1) predicts adverse outcomes: A new candidate docetaxel resistance gene for prostatic cancer (PCa). Bioengineered.

[13] Codreanu S.G., Hoeksema M.D., Slebos R.J.C., Zimmerman L.J., Rahman S.M.J., Li M., Chen S.-C., Chen H., Eisenberg R., Liebler D.C. (2017). Identification of Proteomic Features To Distinguish Benign Pulmonary Nodules from Lung Adenocarcinoma. J. Proteome Res..

[14] Wang Y. (2021). circ-ANXA7 facilitates lung adenocarcinoma progression via miR-331/LAD1 axis. Cancer Cell Int..

[15] Leng X., Wei S., Mei J., Deng S., Yang Z., Liu Z., Guo C., Deng Y., Xia L., Cheng J. (2021). Identifying the prognostic significance of B3GNT3 with PD-L1 expression in lung adenocarcinoma. Transl. Lung Cancer Res..

[16] Wu Y., Luo J., Li H., Huang Y., Zhu Y., Chen Q. (2022). B3GNT3 as a prognostic biomarker and correlation with immune cell infiltration in lung adenocarcinoma. Ann. Transl. Med..

[17] Xu J., Guo Z., Yuan S., Li H., Luo S. (2022). Upregulation of B3GNT3 is associated with immune infiltration and activation of NF-κB pathway in gynecologic cancers. J. Reprod. Immunol..

[18] Kong K., Zhao Y., Xia L., Jiang H., Xu M., Zheng J. (2021). B3GNT3: A prognostic biomarker associated with immune cell infiltration in pancreatic adenocarcinoma. Oncol. Lett..

[19] Bartha A., Gyorffy B. (2021). TNMplot.com: A Web Tool for the Comparison of Gene Expression in Normal, Tumor and Metastatic Tissues. Int. J. Mol. Sci..

[20] Chandrashekar D.S., Karthikeyan S.K., Korla P.K., Patel H., Shovon A.R., Athar M., Netto G.J., Qin Z.S., Kumar S., Manne U. (2022). UALCAN: An update to the integrated cancer data analysis platform. Neoplasia.

[21] Okayama H., Kohno T., Ishii Y., Shimada Y., Shiraishi K., Iwakawa R., Furuta K., Tsuta K., Shibata T., Yamamoto S. (2012). Identification of genes upregulated in ALK-positive and EGFR/KRAS/ALK-negative lung adenocarcinomas. Cancer Res..

[22] Nagy A., Munkacsy G., Gyorffy B. (2021). Pancancer survival analysis of cancer hallmark genes. Sci. Rep..

[23] Subramanian A., Tamayo P., Mootha V.K., Mukherjee S., Ebert B.L., Gillette M.A., Paulovich A., Pomeroy S.L., Golub T.R., Lander E.S. (2005). Gene set enrichment analysis: A knowledge-based approach for interpreting genome-wide expression profiles. Proc. Natl. Acad. Sci. USA.

[24] Liu C.J., Hu F.F., Xia M.X., Han L., Zhang Q., Guo A.Y. (2018). GSCALite: A web server for gene set cancer analysis. Bioinformatics.

[25] Zhou Y., Zhou B., Pache L., Chang M., Khodabakhshi A.H., Tanaseichuk O., Benner C., Chanda S.K. (2019). Metascape provides a biologist-oriented resource for the analysis of systems-level datasets. Nat. Commun..

