# Supplementary figures and images for: Ladinin 1 Shortens Survival via Promoting Proliferation and Enhancing Invasiveness in Lung Adenocarcinoma
Source: Int J Mol Sci. 2022 Dec 27;24(1):431. doi: 10.3390/ijms24010431 (PMC9820746; doi:10.3390/ijms24010431)

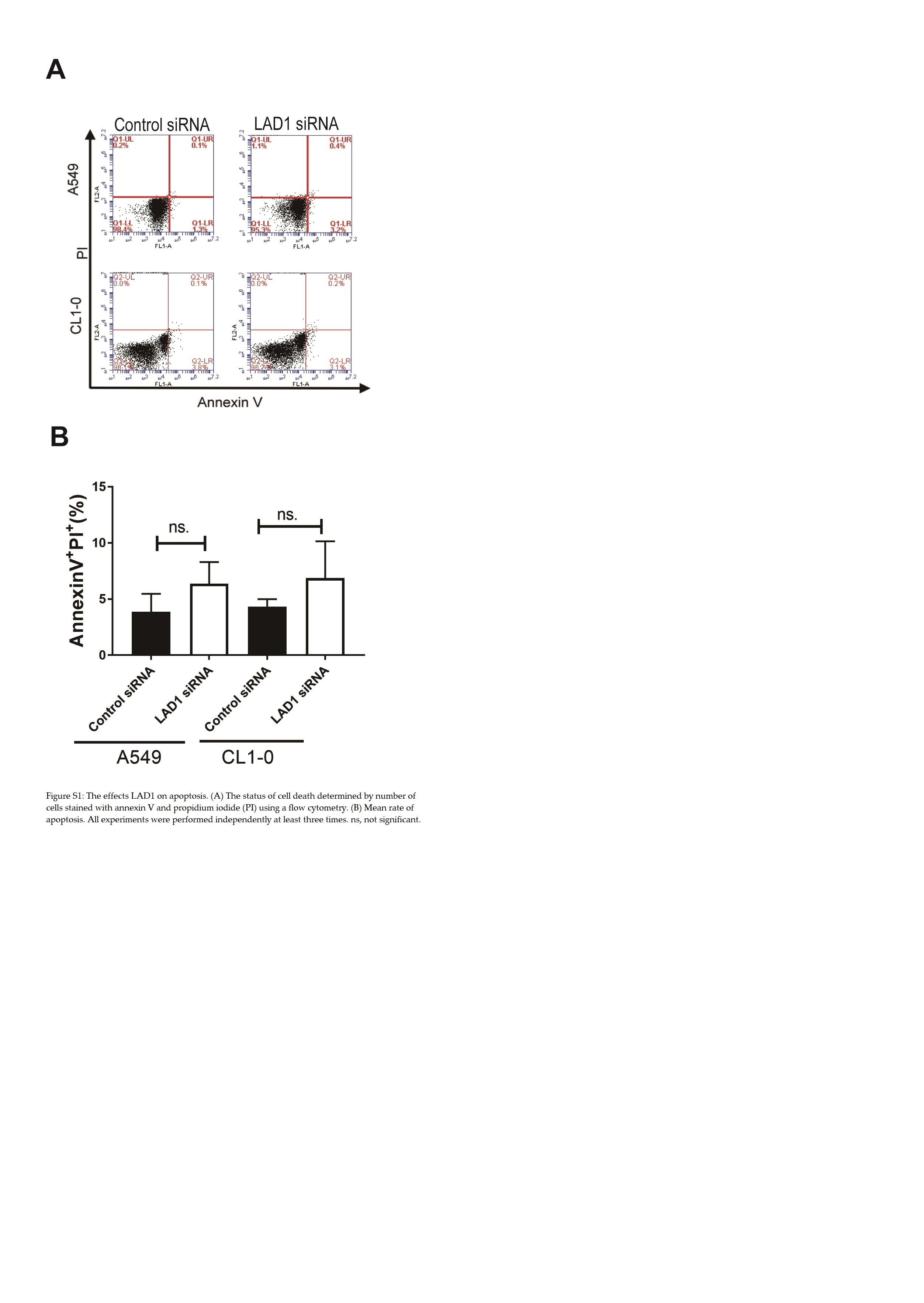

Supplement: Supplementary file 1 [file ijms-24-00431-s001.zip › ijms-2026829-supplementary.jpg]
